# Inflammation and all-cause mortality in patients undergoing peritoneal dialysis

**DOI:** 10.31744/einstein_journal/2024AO0627

**Published:** 2024-07-12

**Authors:** Wander Valadares de Oliveira, Luciane Teixeira Passos Giarola, Letícia Gonçalves Resende Ferreira, Isabella Viana Gomes Schettini, Sylvia Dias Turani, Arlindo Ribeiro de Oliveira, Maria Aparecida Silva Marinho, Sérgio Wyton Lima Pinto, Melina Barros-Pinheiro, Roberta Carvalho de Figueiredo, Danyelle Romana Alves Rios

**Affiliations:** 1 Universidade de São João del-Rei Divinópolis MG Brazil Universidade de São João del-Rei, Divinópolis, MG, Brazil.; 2 Department of Mathematics and Statistics Universidade de São João del-Rei São João del-Rei MG Brazil Department of Mathematics and Statistics, Universidade de São João del-Rei, São João del-Rei, MG, Brazil.; 3 Nephrology Center Complexo de Saúde São João de Deus Divinópolis MG Brazil Nephrology Center, Complexo de Saúde São João de Deus, Divinópolis, MG, Brazil.

**Keywords:** Peritoneal dialysis, Cytokines, Peritoneum, Membranes, Mortality, Inflammation

## Abstract

**Objective:**

This study aimed to evaluate inflammatory biomarkers in patients undergoing peritoneal dialysis and investigate their association with all-cause mortality or transfer to hemodialysis.

**Methods:**

This prospective cohort study included 43 patients undergoing peritoneal dialysis. Plasma levels of cytokines were measured using flow cytometry and capture enzyme-linked immunosorbent assay. Biomarkers were categorized based on their respective median values. Survival analysis was conducted using the Kaplan-Meier estimator, considering two outcomes: all-cause mortality and transfer to hemodialysis.

**Results:**

After adjusting for confounding factors, plasma levels above the median of the levels of CCL2 and plasma, as well as below the median of TNF-α, and the median of dialysate IL-17 levels, were associated with an increased risk of experiencing the specified outcomes after approximately 16 months of follow-up.

**Conclusion:**

These findings suggest that inflammatory biomarkers may be a valuable tool for predicting all-cause mortality and transfer to hemodialysis in patients undergoing peritoneal dialysis.

## In Brief

This prospective cohort study, which included 43 patients undergoing peritoneal dialysis, demonstrates that higher plasma levels of CCL2, lower plasma levels of TNF-α, and lower levels of IL-17 in the dialysate, are associated with a greater risk of transfer to hemodialysis or mortality. These findings emphasize the importance of considering local inflammation, as indicated by cytokine levels, in the risk assessment of patients undergoing peritoneal dialysis, particularly for mortality.

## Highlights

■ Higher plasma levels of CCL2 and lower plasma levels of TNF-α are associated with a greater risk of transfer to hemodialysis or mortality.

■ Lower levels of IL-17 in the dialysate are associated with a greater risk of transfer to hemodialysis or mortality.

■ These findings suggest that inflammatory biomarkers can be valuable tools for predicting all-cause mortality and transfer to hemodialysis in patients undergoing peritoneal dialysis.

## Graphical abstract

**Figure:**
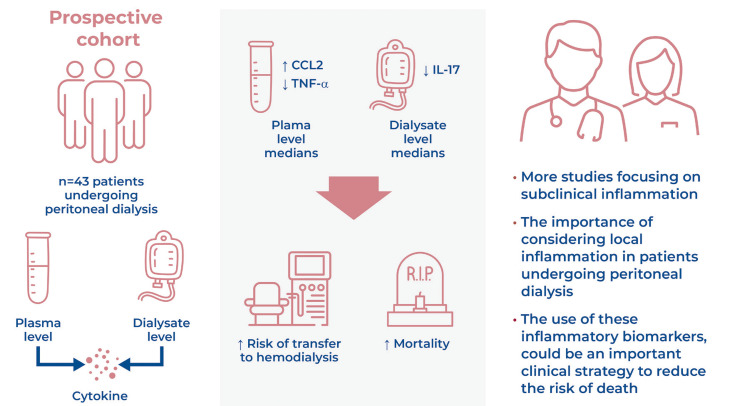

## INTRODUCTION

Peritoneal dialysis (PD) is a renal replacement therapy technique that utilizes the peritoneum, a membrane located within the abdomen that covers the internal organs, to perform blood filtration.^(1,2)^ Patients undergoing PD experience chronic intraperitoneal inflammation that can contribute to the accelerated progression of atherosclerosis.^(3)^ According to the annual census conducted by the Brazilian Society of Nephrology (SBN), the number of patients receiving dialysis for chronic kidney disease (CKD) in the Brazilian public health service has been steadily increasing. Between 2000 and 2021, there was an average annual growth rate of 4.1%, resulting in 148,363 patients in 2021. Among these patients, 5.9% underwent peritoneal PD, with 0.8% receiving continuous ambulatory PD (CAPD), 5.0% undergoing automatic PD (APD), and 0.1% receiving intermittent PD (IPD).^(4,5)^

Over time, patients undergoing PD may develop comorbidities or complications that necessitate switching to hemodialysis (HD), which is the primary alternative therapy. Within the first two years of PD treatment, approximately 20% of patients are transferred to HD for various reasons such as catheter-related complications, peritonitis, infections, or loss of peritoneal function. These factors contribute to the need for the transition to HD as a renal replacement therapy option for these patients.^(6)^ The mortality rate in patients with CKD undergoing PD treatment is 6.1-7.8 times higher than that in the general population of the same age, and cardiovascular diseases (CVD) are the main causes of these deaths.^(7)^

Although the main causes of CKD are systemic arterial hypertension (SAH) and *diabetes mellitus* (DM), CVD-related mortality in patients cannot be solely explained by these traditional factors. Most patients undergoing PD use bioincompatible dialysis solutions with high glucose concentrations, which can stimulate a proinflammatory environment and have been associated with CVD.^(8)^ However, some authors have reported that the use of biocompatible dialysis solutions does not result in a significant improvement in cardiovascular risk among these patients.^(9-11)^ Furthermore, it is well documented that patients with CKD have a heightened risk of infection and increased mortality rates, as compared to individuals without CKD who experience severe infectious episodes.^(12,13)^

The use of inflammatory biomarkers in clinical practice appears to be beneficial for the diagnosis and prognosis of issues associated with PD as it assists in identifying patients who are at a higher risk of complications related to the technique and those who may require closer monitoring and targeted interventions to mitigate the potential complications associated with PD therapy.^(11,14)^ In this context, the identification of these risk factors is crucial to facilitate improved prevention and intervention strategies for the management of patients with CKD undergoing dialysis.^(15)^

Several inflammatory biomarkers have been suggested in the investigation of the prediction of death, despite the risk of CVD.^(16,17)^ In particular, inflammatory markers, such as interleukin (IL), IL-6, IL-10, IL-17, interferon-gamma (IFN-γ), tumor necrosis factor-alpha (TNF-a), and the C-C Motif Chemokine Ligand 2 (CCL2).

Although local peritoneal inflammation is considered a potential contributor to systemic inflammation, the analysis of inflammatory biomarkers and their impact on clinical outcomes, including the risk of mortality, remains to be further elucidated in patients undergoing PD.^(9,18)^

## OBJECTIVE

To evaluate inflammatory biomarkers in patients undergoing peritoneal dialysis and investigate their association with the occurrence of death or transfer of treatment to hemodialysis.

## METHODS

### Study design and population

This prospective cohort study included patients undergoing PD at the Nephrology Center of the *Complexo de Saúde São João de Deus,* Divinópolis, MG, Brazil. At the beginning of this study (August 2011), 296 patients (222 on HD and 74 on PD) were treated at one of the largest nephrology centers in Brazil. All patients underwent Continuous Ambulatory PD/Automated PD (CAPD/APD) as a PD modality.

All eligible patients were invited to participate in the study after a routine outpatient visit which was held once a month. Of the 74 patients who underwent PD, 43 were considered eligible based on the previously established inclusion and exclusion criteria. Inclusion criteria were: being on PD for at least 90 days and being 18 years of age or older. The exclusion criteria were having acute diseases, having autoimmune diseases, having neoplasms, being HIV-positive, having had an episode of peritonitis one month before and/or one month after the evaluation, pregnancy, and being unable to sign the Free and Informed Consent Form (FICF) due to psychiatric illness or mental disorder.

**Figure 1:**
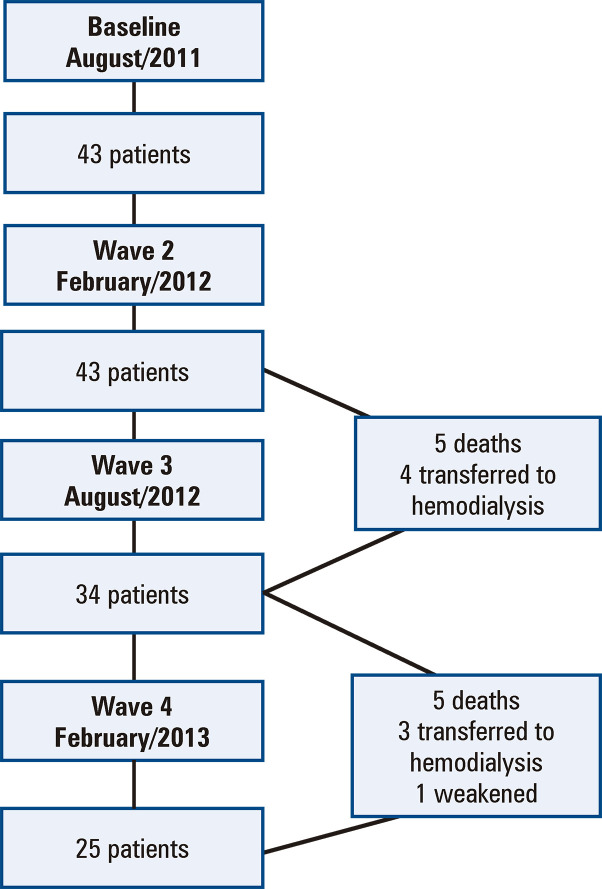
Flowchart of the population in each of the stages of the study

A total of 43 patients participated in the baseline study (August 2011). Wave 2 occurred in February 2012, with no losses during follow-up. In Wave 3, which occurred in August 2012, there were nine losses (20.9%), with five patients due to deaths (11.6%) and four being transferred to HD (9.3%). Finally, wave 4 occurred in February 2013, and during the follow-up, we had nine more losses (20.9%), with five deaths (11.6%); three being transferred to HD (7.0%), and one patient being debilitated (2.3%), thus ending the study with 25 patients (Figure 1).

### Data collection

Data collection was performed at the time of routine consultations, which took place every six months. Biological samples (blood and dialysate) were collected and patient charts were analyzed. Information was collected on sociodemographic data [gender (male; female) and age (in years)]; health conditions [body mass index (BMI), systolic and diastolic blood pressure, primary cause of CKD, DM (yes; no)], laboratory test (serum creatinine), medication use [use of antihypertensive drugs (yes; no), antidiabetics (yes; no) and statins (yes; no)], number of occurrences of peritonitis, complications, total length of stay for PD and date of death.

A total of 5mL of venous blood was collected from all participants using polyethylene syringes and transferred to tubes containing the anticoagulant ethylenediaminetetraacetic acid (EDTA). The samples were centrifuged at 3,500rpm, at room temperature, for 15 minutes, in a Novatecnica^®^ centrifuge model NT815, to obtain plasma. Plasma was aliquoted into Eppendorf^®^ tubes and stored at -80^o^C until measurements were taken. Approximately 10mL of dialysate was collected by draining the dialysis bath by gravity during time 0 of the Peritoneal Equilibration Test (PET) using a sterile bottle. These samples were later aliquoted into Eppendorf^®^ tubes and stored at -80^o^C until the measurements were performed.

### Study variables

#### Outcomes

**Total deaths and transfer of treatment to hemodialysis**

Data on all-cause mortality and transfer of treatment to HD up to February 2013 were collected from the medical records of patients and checked using the Nefrodata computer system of the Nephrology unit.

#### Exposure

**Plasma and dialysate levels of cytokines**

Cytokines (IL-2, IL-4, IL-6, IL-10, IL-17, TNF-a, and IFN-γ) were determined using the Cytometric Bead Array TM kit, catalog number: 560484 (CBA; BD Biosciences, San Jose, CA). Plasma and dialysate samples were mixed with capture beads for each cytokine. After mixing, anti-IL2, anti-IL4, anti-IL-6, anti-IL-10, anti-IL-17, anti-IFN-γ, and anti-TNF-a human antibodies were conjugated to phycoerythrin (PE) and incubated for 2 hours at room temperature, while protected from light. The tubes were then centrifuged (200g for 5 minutes) and the supernatants were carefully aspirated and discarded. The pellets containing the beads were resuspended and the samples were analyzed using the BD LSR Fortessa cytometer (BD Company, San Diego, CA). The data obtained was analyzed using the BD™ Cell Quest and FCAP Array software. The tests were performed in a hematology laboratory at the *Universidade Federal de Minas Gerais* (UFMG). Plasma levels of IL-2 and IL-4 and levels of IL-2, IL-4, and TNF-a in dialysate were not used, as all results were equal to 0 or there was no reading by the equipment. The theoretical limit of detection for each cytokine using these kits is defined as the corresponding concentration at two standard deviations above the median fluorescence of 30 replicates of the negative control (0 pg/mL), the minimal limit of detection data is: IL-2 - 2.6pg/mL, IL-4 - 4.9pg/mL, IL-6 - 2.4pg/mL, IL-10 - 4.5pg/mL, IL-17 - 18.9pg/mL, TNF-a - 3.8pg/mL, and IFN-γ - 3.7pg/mL.

#### Plasma and dialysate levels of CCL2 (pg/mL)

CCL2 levels were measured using capture enzyme-linked immunosorbent assay capture (ELISA) (catalog number: ABIN6954594; R&D Systems, Minneapolis, MN, USA) as per the instructions of the manufacturer. The reactions were read using a VersaMax Microplate Reader (Molecular Devices, USA). The reference range and intra- and inter-assay coefficients of variation for plasma CCL2 provided by the manufacturer were 134-436pg/mL, 5.8%, and 5.7%, respectively. The detection range of the kit was 15.6pg/mL - 1000pg/mL. Patients were categorized into two groups: those with values less than or equal to the median were classified as 0, while those with values greater than the median were classified as 1.

#### Confounding factors

Factors that, according to the scientific literature, would be associated with exposure and outcome were considered confounders, namely: sex, age, systolic blood pressure, serum creatinine levels, occurrence of peritonitis (whether or not there was peritonitis during the study), the use of HD before PD, use of antihypertensives, antidiabetics, and statins, as well as total time on PD (≤ and >3 years of treatment).

## Statistical analysis

Biomarkers (IL-6, IL-10, IL-17, TNF-a, INF-γ, and CCL-2 in plasma; IL-6, IL-10, IL-17, and CCL2 in dialysate) were categorized by their respective medians (Table 1): values equal to or less than the median were assumed to be zero and higher values were assumed to be 1.

**Table 1:** Median of the studied biomarkers

	Median (pg/mL)
Plasma biomarkers	
IL-6	8.29 (5.48-15.55)
IL-10	2.58 (1.54-3.06)
IL-17	7.78 (5.02-12.96)
TNF-a	2.23 (1.24-2.84)
INF-γ	3.75 (2.17-4.65)
CCL2	175 (120.0-245.5)
Biomarkers in dialysate	
IL-6	29.79 (8.6-87.09)
IL-10	0.96 (0.67-1.17)
IL-17	11.84 (5.97-18.01)
CCL2	266.8 (114.6-448.0)

Results are expressed in medians (interquartile ranges).

IL: interleukin; TNF-a: tumor necrosis factor-alpha; IFN-γ: interferon-gamma; CCL2: (*C-C Motif Chemokine Ligand 2*).

Statistical analysis began by estimating the survival of individuals using the Kaplan-Meier estimator. Subsequently, to identify the techniques and models suitable for the analysis of patient lifespans, it was necessary to observe the characteristics of these patients.

Each patient is not only at risk of death but also at risk of transferring treatment to HD, *e.g.*, due to the degradation of the peritoneal membrane (PM). This transfer took place with six patients, as mentioned above, and thus, their follow-ups were lost. Although patients transferred to HD are at risk of death, only those on PD were analyzed, and patients transferred to HD were removed from this group.

Two outcomes were considered in the model: death and the transfer of treatment to HD. In this study, the time until the occurrence of one of these events, whichever occurred first, was computed, and the events of death and transfer of treatment to HD were treated as competitive. If none of the outcomes occurred in a given individual, their lifetime was considered censored. Thus, the failure indicator variable is represented by:


$$\delta_{i}=\left\{ \begin{array}{c} 1,\text{ if }t_{i}\text{ is a time of death or transfer to HD }\\ 0,\text{ if }t_{i}\text{ is the time due to the end of the study (censorship) } \end{array}\right.$$

Owing to the competitive risk approach, the analysis strategy followed the context of marginal models for such risks, which present a characterization analogous to the classic Cox regression model.^(19)^ Thus, to investigate whether the levels of each cytokine in the plasma and dialysate interfered with the risk of failure and considering all the variables evaluated at the beginning of the follow-up, we adjusted the model:


$$\lambda_{i}(t)=\lambda_{0}(t)\exp\left\{\sum_{j=1}^{12}\beta_{j}x_{ij}\right\},i=1,\ldots ,43$$

The parameter being associated with the *j*-th covariate, the observed value of the *j*-th covariate for the *j*-th individual, and the baseline risk.

The variable selection process was performed using the Akaike Information Criterion (AIC). The selection of complete and restricted models was followed by a Likelihood Ratio Test (TRV). The Wald Test was used to investigate the significance of the variables.^(20)^ All tests were performed at a 5% significance level. Variables related to the time on PD, sex, age, number of episodes of peritonitis, and previous HD treatment were considered in the models, although they were not significant because of their importance in the study. The parameters of the models, which refer to the common (average) effect of the variables on the two outcomes, were estimated using the maximum partial likelihood.

The proportional hazard assumption was verified through a graphic evaluation of the Schoenfeld residuals, which indicated a violation of the proportionality of risks if trends occurred over time. Pearson’s correlation coefficient between these residuals and time was also obtained, and a test was performed under the null hypothesis of no correlation. Thus, the low probability of significance indicates a violation of the assumption of risk proportionality.

The adequacy of the adjusted model was based on Martingale and Deviance residuals, which allowed us to identify atypical individuals (outliers). The martingale residues range from (-∞,1) and must be randomly distributed around zero if the model is suitable. Deviance residuals must also present random behavior around zero, but they are acceptable within the range (-3,3).

All analyzes were carried out in the R (2021) software with the aid of packages ‘Survival’^(21)^ and ‘Mass’.^(22)^

## Ethical aspects

This study was approved by the Research Ethics Committee of *Universidade Federal de São João Del-Rei:* CAAE: 19284613.5.0000.5545; # 462.569. and *Hospital São João de Deus*: CAAE: 19284613.3.3001.5130; # 4.063.067.

## RESULTS

The demographic and clinical characteristics of patients at baseline are shown in table 2. Overall, most participants were male (51.2%) and had a mean age of 63.0 (SD=15.3) years. The average BMI was 24.5 (SD=4.4), and 58.5% of the patients had DM. The mean systolic blood pressure was 142.0 (SD=21.4) mmHg, and the mean serum creatinine levels were 9.6 (SD=4.3) mg/dL. The most prevalent primary disease was diabetic kidney disease (30.2%), followed by hypertensive nephrosclerosis (23.2%). The most commonly used antihypertensive medications were diuretics (81.4%), followed by β-blockers (51.2%), and angiotensin receptor antagonists (ARA) (48.8%). In addition, 30.2% of the participants used insulin, 44.2% used oral antidiabetic agents, and 53.5% used statins.

**Table 2:** Distribution of the study population at the baseline according to demographic and clinical characteristics

Variables	(n=43)
Age (Years)	63.0 (15.3)
Sex	
Male, n (%)	22 (51.2)
Primary causes of CKD, n (%)	
Diabetes kidney disease	13 (30.2)
Hypertensive nephrosclerosis	10 (23.2)
Chronic glomerulonephritis	8 (18.6)
PKD, CAKUT, and obstructive uropathy	6 (13.9)
Unknown etiologies	6 (13.9)
Blood pressure	
Systolic pressure (mmHg)	142.0 (21.4)
Diastolic pressure (mmHg)	82 (80-90)
Diabetes, n (%)	24 (58.5)
BMI (kg/m^2^)	24.5 (4.4)
Serum creatinine (mg/dL)	9.6 (4.3)
Medication, n (%)	
β-blockers	22 (51.2)
ARA	21 (48.8)
Calcium channel antagonists	17 (39.5)
ACE inhibitors	2 (4.7)
Diuretics	35 (81.4)
Anxiolytics/Antidepressants	16 (37.2)
Vitamin supplements	15 (34.9)
Acetylsalicylic acid	20 (46.5)
Statins	23 (53.5)
Oral antidiabetics	19 (44.2)
Insulin	13 (30.2)

BMI: body mass index; CKD: chronic kidney disease; PKD: polycystic kidney disease; CAKUT: congenital anomalies of the kidney and urinary tract; PD: peritoneal dialysis; ARA: angiotensin receptor antagonist; ACE: angiotensin-converting enzyme inhibitor.

Results are presented as the mean and standard deviation for data with a symmetrical distribution and median (interquartile range) for those with a skewed distribution. Categorical variables are presented as proportions (n [%]).

Of the 43 patients at baseline, 10 (23.3%) died during the 18-month follow-up period. The first death occurred after 7 months from the beginning of the study, and the last one after 17.8 months, with a mean follow-up time of 16.4 (± 4.0) months. Among these, five (50%) had a total PD treatment time of ≤3 years (Table 3). The causes of mortality were attributed to the following factors: sepsis (40%), undetermined causes (20%), pleural infection (10%), postoperative complications (10%), severe anemia (10%), and acute myeloid leukemia (10%). During the study, seven patients were transferred to HD, primarily due to peritoneal membrane failure (57.1%), recurrent peritonitis (28.6%), and catheter-related infections (14.3%).

**Table 3:** Characteristics of the study population according to overall mortality

Variables	Patients	
	No deaths Group (n=33)	Deaths Group (n=10)
Age (Year)	61.0 (16.2)	68.0 (10.8)
Sex		
Male, n (%)	17 (51.5)	5 (50)
Blood pressure		
Systolic pressure (mmHg)	144.0 (21.6)	136.0 (20.7)
HD before PD, n (%)	8 (24.2)	2 (20)
Occurrence of peritonitis, n (%)	12 (36.4)	3 (30)
Medication use		
Antihypertensives	17 (51.5)	5 (50)
Antidiabetics	10 (30.3)	7 (70)
Time of PD ≤ 3 years	11 (33.3)	5 (50)

Baseline patients: n=43. Nonparametric variables are presented as medians (interquartile ranges). Categorical variables are presented as proportions (n [%]).

HD: hemodialysis; PD: peritoneal dialysis.

**Figure 2:**
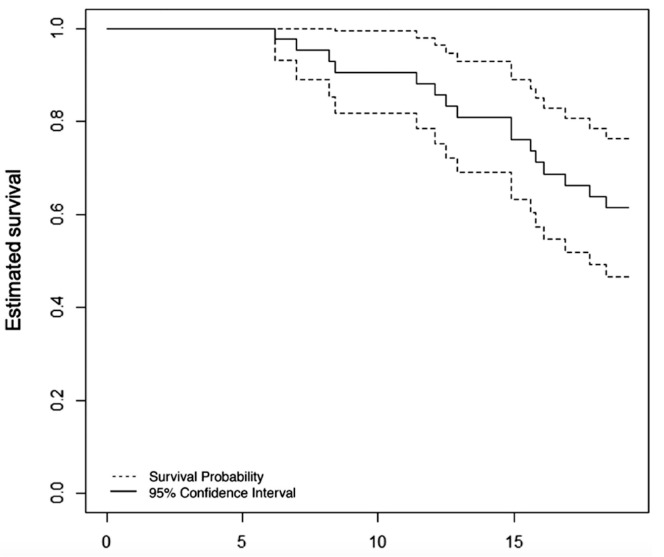
Point and 95% interval estimates for survival, obtained from the Kaplan Meier estimator for the 43 patients in the study

Figure 2 displays the survival estimates and their corresponding 95% confidence intervals calculated using the Kaplan-Meier estimator. It is evident from the graph that the survival estimates exhibited high values, surpassing 0.7. This can be attributed to the substantial number of censorships (27 individuals) that occurred because of the relatively short follow-up period of approximately 18 months. The specific values can be found in the Supplementary Material, Table 1S.

In Cox regression modeling with competitive risks, considering plasma levels, cytokines IL-6, IL-10, IL-17, and INF-γ showed no statistically significant association with the outcome at the 5% level, and thus, no model was adjusted. However, upon analyzing the cytokines TNF-a and CCL2, such associations were observed (Tables 4 and 5).

**Table 4:** Estimates obtained for the parameters of the adjusted Cox model, and 95%CI upon considering the competitive risks of death, transfer to hemodialysis, and the TNF-α cytokine levels in plasma were categorized according to the median

Parameter	Coefficient	Exponential (Coef)	Standard Error (Coef)	z	Pr (>|z|)	95%CI (RR)
PD time	-0.018	0.982	0.009	-1.874	0.060	[0.963; 1.000]
Sex	-0.455	0.634	0.571	-0.798	0.425	[0.206; 1.942]
Age	0.005	1.005	0.019	0.290	0.771	[0.968; 1.043]
HD prior to PD	-0.757	0.469	0.703	-1.075	0.282	[0.118; 1.864]
Number of peritonitis	0.854	2.350	0.323	2.642	0.008*	[1.246; 4.430]
Plasma TNF-a	-1.191	0.303	0.591	-2.015	0.043*	[0.095; 0.968]

* Significant at 5%.

PD: peritoneal dialysis; HD: hemodialysis; TNF-a: tumor necrosis factor-alpha.

**Table 5:** Estimates obtained for the parameters of the adjusted Cox model and 95%CI, considering the competitive risks of death, transfer to hemodialysis, and the CCL2 cytokine levels in plasma were categorized according to the median

Parameter	Coefficient	Exponential (Coef)	Standard Error (Coef)	z	Pr (>|z|)	95%CI (RR)
PD time	-0.022	0.978	0.010	-2.055	0.039*	[0.957; 0.999]
Sex	-0.507	0.602	0.574	-0.883	0.377	[0.195; 1.857]
Age	0.018	1.018	0.018	0.964	0.334	[0.981; 1.056]
HD prior to PD	-0.585	0.556	0.697	-0.840	0.400	[0.142; 2.183]
Number of peritonitis	0.752	2.123	0.318	2.360	0.018*	[1.136; 3.967]
Plasma CCL2	1.430	4.182	0.643	2.224	0.026*	[1.185; 14.761]

* Significant at 5%.

PD: peritoneal dialysis; HD: hemodialysis; CCL2: (C-C Motif Chemokine Ligand 2).

Regarding the dialysate, cytokines IL-6, IL-10, TNF-a, INF-γ, and CCL2 did not exhibit a statistically significant association with the outcome at the 5% significance level. Consequently, no model was adjusted for these cytokines. However, upon analyzing IL-17, significant associations were observed, and the model was adjusted for the number of peritonitis occurrences, IL-17 levels, and other relevant variables considered in the study.

The results of the hypothesis test for the Pearson correlation coefficient between the Schoenfeld residuals and time indicated that there was no violation of the proportional hazard assumption. The p-values obtained for both the global test and each covariate were >5%. These results are presented in detail in the Supplementary Material, Tables 2S and 3S. Figures 1S, 2S, 3S and 4S. Additionally, the estimates of the parameters of the adjusted Cox model and the corresponding 95%CI, while considering the competitive risks of death and transfer to HD, are provided in the Supplementary Material, Tables 2S, 3S, 4S and 5S, and Figures 5S and 6S. These estimates pertain to the cytokines CCL2 and IL-17, which were categorized by the median in the dialysate.

## DISCUSSION

This prospective cohort study investigated the association between inflammatory biomarkers and the occurrence of all-cause mortality and transfer to HD in patients undergoing PD. The study findings revealed that, after adjusting for relevant factors, elevated plasma levels of CCL2 above the median, decreased plasma levels of TNF-a, and decreased IL-17 dialysate levels below the median were associated with a higher risk of experiencing the defined outcome. These associations were observed after a follow-up period of approximately 16 months.

Mortality in patients undergoing PD is 6.1 to 7.8 times higher than that in the general population of the same age, even exceeding the numbers that occur in neoplastic diseases. Cardiovascular diseases remains the leading cause of death in these patients.^(7)^ In addition, several studies have shown that peritonitis, one of the main infectious and inflammatory processes affecting patients undergoing PD, is related to an increased all-cause mortality rate in this population.^(3,23-26)^

The identification of inflammatory biomarkers plays a crucial role in the management of patients undergoing PD as they can contribute to prolonging therapy duration and reducing the risk of mortality. One such biomarker is TNF-a, which exerts diverse cellular effects. At lower concentrations, TNF-a acts as a paracrine or autocrine regulator of leukocytes and endothelial cells, playing a significant role in the modulation of the inflammatory response. A study conducted with animal models demonstrated that mice lacking the p55 TNF-a receptor were highly susceptible to certain bacterial infections, highlighting the importance of TNF-a in the immune response.^(27,28)^

Indeed, even at low concentrations, TNF-a increases the chemotaxis of macrophages and neutrophils, as well as enhances their phagocytic and cytotoxic activities.^(29)^ This promotes leukostasis, which refers to the accumulation of white blood cells in the microvasculature, leading to target organ complications such as microvascular leukoaggregates, hyperviscosity, tissue ischemia, infarction, and hemorrhage. These complications were not attributable to infection, thromboembolism, or other underlying causes. Additionally, TNF-a induces increased expression of intracellular adhesion molecule-1 (ICAM-1) and vascular cell adhesion molecule-1 (VCAM-1) at the sites of inflammation.^(30,31)^

The findings of the present study are in agreement with those of a study by Janda et al. that followed 55 patients undergoing PD treatment over six years. The study concluded that higher levels of TNF-a was significantly associated with all-cause and cardiovascular mortality, independent of other factors.^(32)^

Some studies have suggested that subclinical inflammation may be an important factor contributing to the presence of low concentrations of inflammatory biomarkers and increased mortality.^(9,33,34)^

Patients undergoing PD have a chronic inflammatory profile, which may be present even subclinically, without affecting all inflammatory biomarkers. However, even at low concentrations, the presence of these biomarkers can negatively affect patient health.^(35)^ This is consistent with the findings of the current study, where lower median plasma levels of TNF-a were associated with significant negative effects on the health of patients undergoing PD, including an increased risk of transferring to HD and mortality.

In contrast, our findings demonstrated that higher median plasma levels of CCL2 were associated with an increased risk of changing dialysis modalities and mortality, regardless of the timeframe. This factor was found to influence the outcomes. Previous studies have also indicated that elevated CCL2 levels are associated with increased cardiovascular mortality in both the general population and patients on dialysis.^(36)^ This biomarker plays a crucial role in the initiation and progression of inflammation.^(37)^

Elevated plasma levels of CCL2 have been found in individuals with classic risk factors for the development of coronary artery disease (CAD), such as advanced age, hypertension, hypercholesterolemia, CKD, and CVD. Increased CCL2 levels are associated with an increased risk of mortality in this population.^(38,39)^ This occurs due to the systemic pro-inflammatory environment, which can result in a higher recruitment of cells, with monocytes and macrophages being the primary cells found at sites of inflammation. This increased cellular activity contributes to the production of inflammatory cytokines including CCL2.^(40)^

Our findings are consistent with those of Ko et al., who followed 169 patients undergoing PD treatment over a period of 4.9 years. The study concluded that CCL2 levels were strongly associated with nutritional and systemic inflammatory markers in patients undergoing PD. Furthermore, elevated CCL2 levels in the dialysate were significantly associated with increased all-cause and cardiovascular mortalities.^(41)^

Piemonti et al. investigated 207 women selected from a population survey conducted between 1990-1991 in Lombardy, Italy (Cremona Study) and found that CCL2 levels at baseline correlated with a greater risk of developing CVD. In addition, in this study, high plasma levels of CCL2 were found to be significantly associated with cardiovascular mortality in a univariate analysis.^(24,42-44)^

Regarding IL-17, our findings indicated that patients with lower levels of this cytokine in the dialysate had a seven-fold higher risk of death than those with levels above the median. IL-17 is typically absent in the peritoneum of healthy individuals but can be readily detected in the peritoneal membrane biopsies and dialysate of patients undergoing PD. Its presence may be triggered by various types of infections and can influence the course of local inflammation, potentially leading to alterations in the peritoneal membrane vasculature and an increased risk of mortality in patients undergoing PD.^(45,46)^

Exposure of the peritoneal membrane to PD solutions induces cellular and molecular responses including inflammation, cell death, phenotypic changes, angiogenesis, and submesothelial collagen accumulation, ultimately leading to peritoneal membrane failure. Local production of interleukin IL-17 in the damaged peritoneum by infiltrating immune cells in the damaged peritoneum can contribute to the amplification of the pro-inflammatory state by recruiting additional inflammatory cells into the peritoneal cavity. This, in turn, increases the potential processes induced by IL-17 in the peritoneum, including angiogenesis, cell differentiation, and fibrosis.^(47)^

IL-17 acts as an adjuvant for other cytokines, such as TNF-a, and positively regulates Th17-related cytokines, like IL-6^(48)^ and IL-1,^(49)^ resulting in synergistic actions that contribute to a pro-inflammatory and pro-fibrotic state within the peritoneal cavity during PD.

Despite this, the role of IL-17 in CVD development and mortality, especially in patients undergoing PD, remains unclear. This may be because of the pleiotropic mechanism of this cytokine, which makes it difficult to interpret and fully elucidate its mechanism of action, as shown by the conflicting results obtained in several studies. Some studies suggest that this cytokine has a proatherogenic role,^(50-54)^ while others suggest an atheroprotective role through cross-regulation with pro-inflammatory cytokines such as IL-6 and INF-g.^(55,56)^

Although there has been increasing attention in recent years to the effects of IL-17 in PD patients, studies evaluating the association between this biomarker and mortality are scarce. Further studies are required to elucidate their roles in patients undergoing PD. To our knowledge, this is the first study conducted in Brazil to investigate the association between IL-17 levels and mortality in patients undergoing PD.

The results of our study contribute to the understanding of the pathophysiological mechanisms and the nature and direction of the effects of risk factors in stratifying cardiovascular risk and predicting mortality. They also shed light on the effects of drugs known to interfere with the inflammatory processes in PD.

However, this study has a few limitations. First, we relied on information from secondary sources, such as patient charts, which may not always be clear and accurate. Additionally, the sample size of our study was small, although it represents a population of 76 patients from one of the largest nephrology centers in Brazil for patients undergoing PD, and is comparable to the sample sizes of other studies.^(57,58)^ If we had a larger sample size, stratifying these patients would have revealed more robust results regarding inflammation and mortality in this population. Another limiting factor was the relatively short follow-up time of the patients as compared to other studies mentioned in our research. A longer follow-up period may have revealed additional associations between local inflammation, systemic inflammation, and mortality.

On the other hand, there are several strengths to highlight in our study. First, this was the first study conducted in Brazil to investigate the association between IL-17 levels and all-cause mortality in patients undergoing PD. The findings of our study provide valuable data regarding biomarkers in patients undergoing PD treatment. Therefore, it is possible to propose more longitudinal studies that evaluate the levels of inflammatory biomarkers in both plasma and dialysates over time as a source of clinical information for patients. Monitoring the levels of these biomarkers in the plasma and dialysate can offer insights into the systemic and local pro-inflammatory states, respectively. The importance of identifying and quantifying these biomarkers lies in their individualized monitoring, as short-term increases in their levels can indicate systemic and local inflammatory processes and an increased risk of developing CVD, and consequently, death.

## CONCLUSION

In conclusion, our study demonstrates that higher plasma levels of CCL2, lower plasma levels of TNF-a, and lower levels of IL-17 in the dialysate are associated with a greater risk of transfer to HD or mortality. These findings emphasize the importance of considering local inflammation, as indicated by cytokine levels, in risk assessments, particularly for mortality, of patients undergoing PD. Additionally, studies focusing on subclinical inflammation, even those with low levels of inflammatory biomarkers, should be considered in the future. Understanding and mitigating the damage caused by early subclinical local inflammation using these inflammatory biomarkers whenever possible could be an important clinical strategy for reducing the risk of death in this population.

## SUPPLEMENTARY MATERIAL

Inflammation and all-cause mortality in patients undergoing peritoneal dialysis


