# Supplementary material for: Inflammation and all-cause mortality in patients undergoing peritoneal dialysis
Source: Einstein (Sao Paulo). 2024 Jul 12;22:eAO0627. doi: 10.31744/einstein_journal/2024AO0627 (PMC11323835; doi:10.31744/einstein_journal/2024AO0627)
Supplement: Supplementary file 1 [file 2317-6385-eins-22-eAO0627-suppl01.pdf]

SUPPLEMENTARY MATERIAL

Inflammation and all-cause mortality in patients undergoing peritoneal dialysis

Wander Valadares de Oliveira Júnior, Luciane Teixeira Passos Giarola, Letícia Gonçalves Resende Ferreira, Isabella Viana Gomes Schettini, Sylvia Dias Turani, Arlindo Ribeiro de Oliveira, Maria Aparecida Silva Marinho, Sérgio Wyton Lima Pinto, Melina Barros-Pinheiro, Roberta Carvalho de Figueiredo, Danyelle Romana Alves Rios

DOI: 10.31744/einstein_journal/2024AO0627

Table 1S shows the survival estimates of the 43 patients on PD, their respective standard errors and 95%CI, obtained by the Kaplan Meier estimator.

Table 1S. Survival estimates by Kaplan Meier

Time	Number risk	Event	Survival	SD	LL (95%CI)	UL (95%CI)
6.2	43	1	0.977	0.0230	0.932	1.000
7.0	41	1	0.953	0.0325	0.889	1.000
8.2	40	1	0.929	0.0395	0.852	1.000
8.4	39	1	0.905	0.0451	0.817	0.994
11.4	38	1	0.881	0.0498	0.784	0.979
12.1	37	1	0.858	0.0538	0.752	0.963
12.5	36	1	0.834	0.0574	0.721	0.946
12.9	35	1	0.810	0.0605	0.691	0.929
14.9	33	2	0.761	0.0660	0.631	0.890
15.6	31	1	0.736	0.0683	0.602	0.870
15.8	30	1	0.712	0.0703	0.574	0.850
16.1	29	1	0.687	0.0720	0.546	0.828
16.9	28	1	0.663	0.0735	0.519	0.807
17.8	27	1	0.638	0.0748	0.492	0.785
18.4	26	1	0.614	0.0758	0.465	0.762

SD: standard deviation; LL: lower limit; UL: upper limit.

The table 2S presents the results of the hypothesis test for Pearson's correlation coefficient between Schoenfeld residuals and time. P-values greater than 5%, both in the global test and in the test for each covariate, indicate that there is no violation of the proportional hazards' assumption. This fact is corroborated by the observation of the graphs in figure 1S, in which there is no evidence of trends and it is noted that the residuals are randomly distributed around zero.

Table 2S. Results of the hypothesis test for Pearson's correlation coefficient between Schoenfeld residuals, considering the plasma TNF-α cytokine level and time

Parameter	χ^2	p valor
PD time	1.8272	0.176
Sex	0.0033	0.856
Age	2.4965	0.114
HD prior to PD	0.0911	0.763
Number of peritonitis	2.8045	0.094
Plasma TNF-α	0.1699	0.680
Global	5.0832	0.533

PD: peritoneal dialysis; HD: hemodialysis; TNF-α: tumor necrosis factor-α.

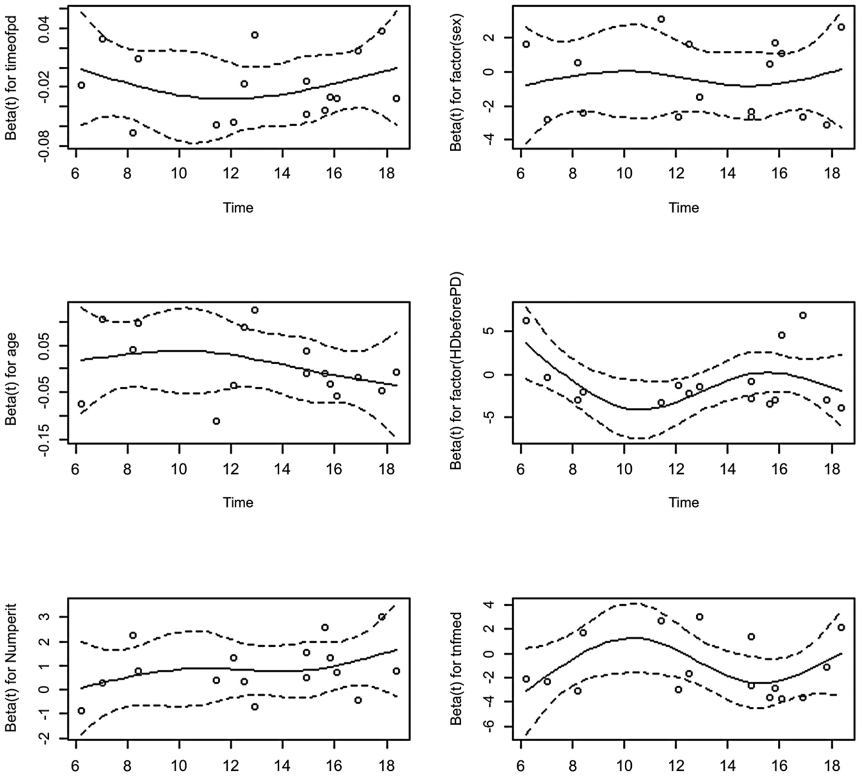

Figure 1S. Plots of Schoenfeld residuals as a function of time for the adjusted Cox model considering the plasma TNF-α cytokine level

The Martingale and Deviance residuals graphs (Figure 2S) constructed to assess the general quality of the fitted model indicate the model's suitability, as the residuals are randomly distributed around zero. Thus, the estimates of the final adjusted model are presented in table 2S, as well as the respective risk ratios. It is possible to conclude that with each occurrence of peritonitis, the risk of death or transfer to HD increases about 2 times. Patients with plasma levels of TNF-α below the median have a 3.29-fold greater risk of death or transfer to HD than patients with TNF-α levels above the median.

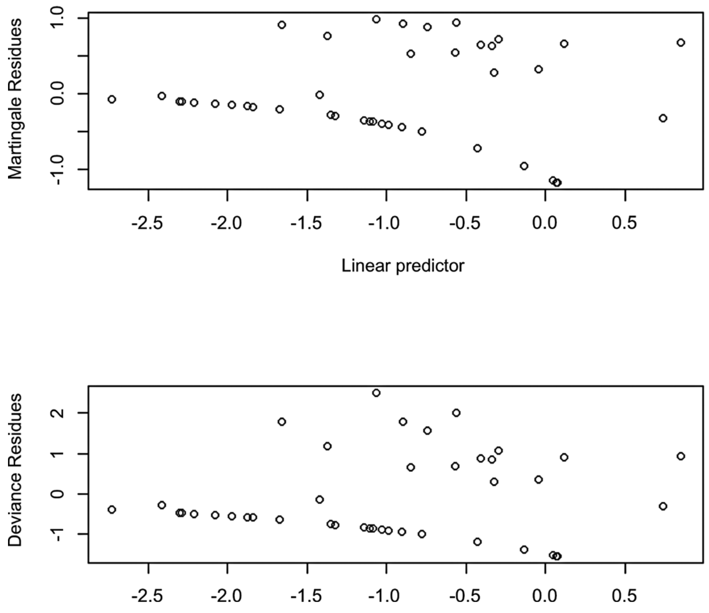

Figure 2S. Martingale and Deviance residues from the Cox model adjusted considering the plasma TNF-α cytokine level

Table 3S. Results of the hypothesis test for Pearson's correlation coefficient between Schoenfeld residuals, considering the plasma cytokine CCL2 level and time

Parameter	χ^2	p valor
PD time	1.8573	0.173
Sex	0.0069	0.933
Age	1.5771	0.209
HD prior to PD	0.0795	0.778
Number of peritonitis	2.7165	0.099
Plasma CCL2	0.0088	0.925
Global	4.2861	0.638

PD: Peritoneal dialysis; HD: Hemodialysis; CCL2: (C-C Motif Chemokine Ligand 2).

The table 3S presents the results of the hypothesis test for Pearson's correlation coefficient between Schoenfeld residuals and time. P-values greater than 5%, both in the global test and in the test for each covariate, indicate that there is no violation of the proportional hazards' assumption. This fact is corroborated by the observation of the graphs in figure 3S, in which there is no evidence of trends and it is noted that the residuals are randomly distributed around zero.

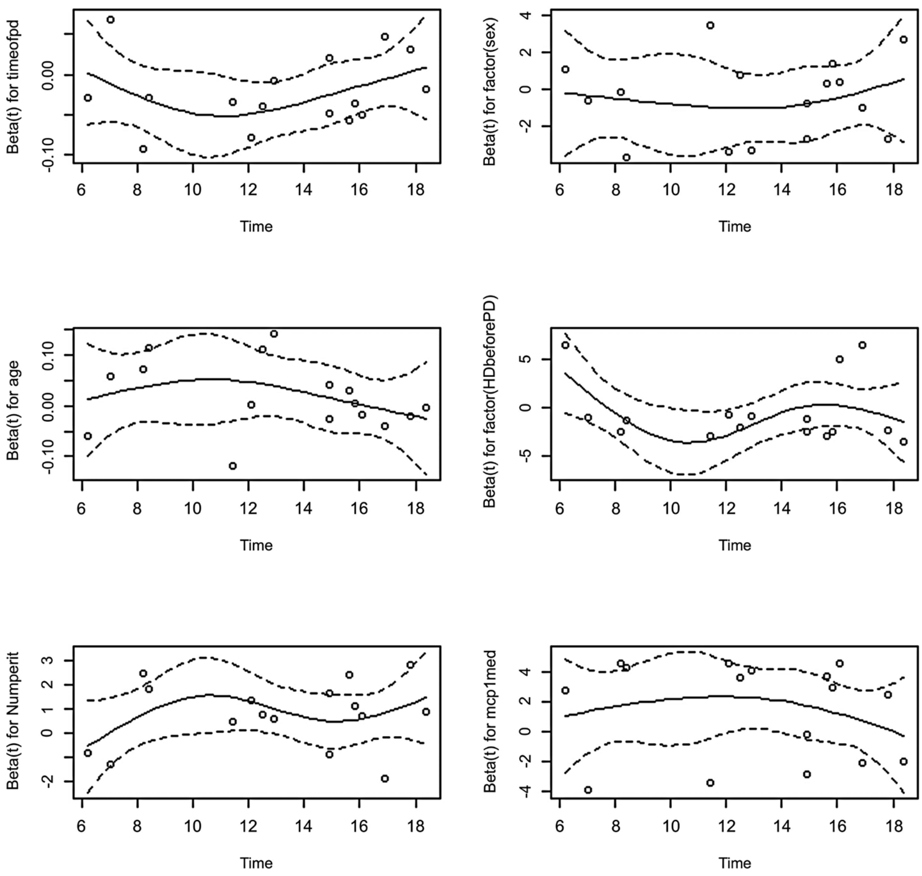

Figure 3S. Graphs of Schoenfeld residuals as a function of time for the adjusted Cox model

The Martingale and Deviance residuals graphs (Figure 4S) constructed to assess the general quality of the fitted model indicate the model's suitability, as the residuals are randomly distributed around zero.

Thus, the estimates of the final adjusted model are presented in table 4S, as well as the respective risk ratios. It is possible to conclude that with each occurrence of peritonitis, the risk of death or transfer to HD increases about 2 times. DP timing does not accelerate or decelerate risk, although it is influential. Patients with plasma CCL2 levels above the median are 4 times more likely to die or transfer to HD than patients with CCL2 levels below the median.

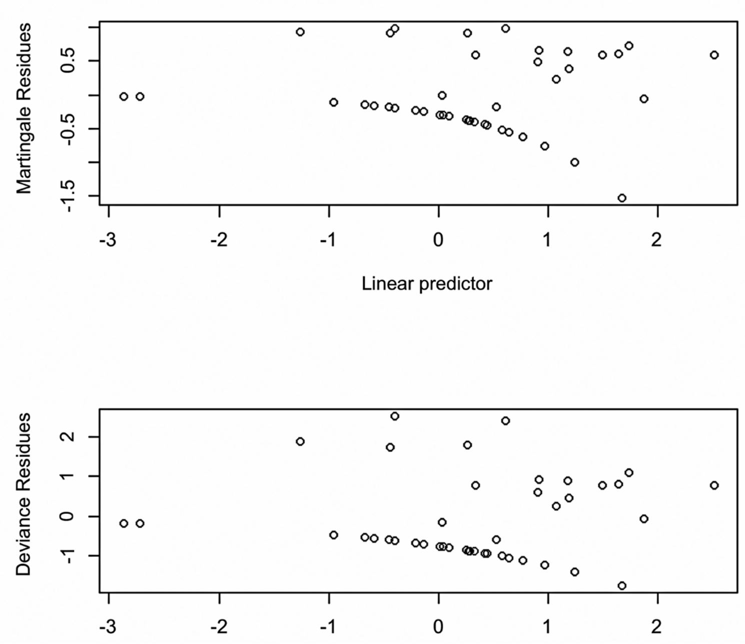

Figure 4S. Martingale and Deviance residues from the Cox model adjusted considering the plasma cytokine CCL2 level

Table 4S. Estimates obtained for the parameters of the adjusted Cox model, and 95%CI, considering the competitive risks of death and transfer to hemodialysis and the cytokine IL-17 categorized by the median in the dialysate

Parameter	Coefficient	Exponential (Coef)	Standard Error (Coef)	z	Pr (>|z|)	95%CI (RR)
PD time	-0.016	0.9984	0.009	-1.582	0.113	[0.965; 1.003]
Sex	-0.800	0.449	0.659	-1.213	0.225	[0.123; 1.636]
Age	-0.002	0.998	0.021	-0.092	0.926	[0.957; 1.040]
HD prior to PD	-0.151	0.860	0.670	-0.224	0.822	[0.231; 3.203]
Number of peritonitis	0.857	2.356	0.353	2.425	0.015*	[1.178; 4.710]
Plasma IL-17	-1.924	0.145	0.726	-2.649	0.008*	[0.035; 0.606]

* Significant at 5%.
PD: peritoneal dialysis; HD: hemodialysis; IL-17: interleukin-17.

The table 5S presents the results of the hypothesis test for Pearson's correlation coefficient between Schoenfeld residuals and time. P-values greater than 5%, both in the global test and in the test for each covariate, indicate that there is no violation of the proportional hazards' assumption. This fact is corroborated by the observation of the graphs in figure 5S, in which there is no evidence of trends and it is noted that the residuals are randomly distributed around zero.

Table 5S. Results of the hypothesis test for Pearson's correlation coefficient between Schoenfeld residuals considering the level of the cytokine IL-17 in the dialysate and time

Parameter	χ^2	p valor
PD time	1.3942	0.240
Sex	0.1055	0.750
Age	2.2997	0.130
HD prior to PD	0.2466	0.620
Number of peritonitis	3.5471	0.060
IL-17 in dialysate	0.0004	0.980
Global	6.3703	0.380

PD: peritoneal dialysis; HD: hemodialysis; IL-17: interleukin-17.

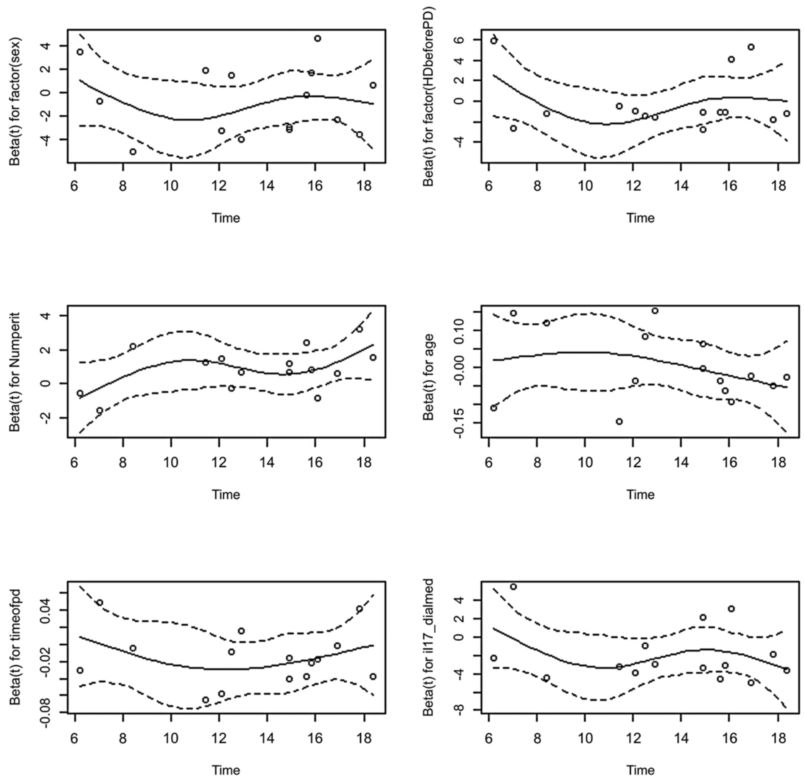

Figure 5S. Plots of Schoenfeld residuals as a function of time for the adjusted Cox model considering the level of the cytokine IL-17 in the dialysate

The Martingale and Deviance residuals graphs (Figure 6S) constructed to assess the general quality of the fitted model indicate the model's suitability, as the residuals are randomly distributed around zero.

Thus, the estimates of the final adjusted model are presented in table 5S, as well as the respective risk ratios. It is possible to conclude that with each occurrence of peritonitis, the risk of death or transfer to HD increases about 2 times. Patients with IL-17 dialysate levels below the median are almost 7 times more likely to die or transfer to HD than patients with IL-17 levels above the median.

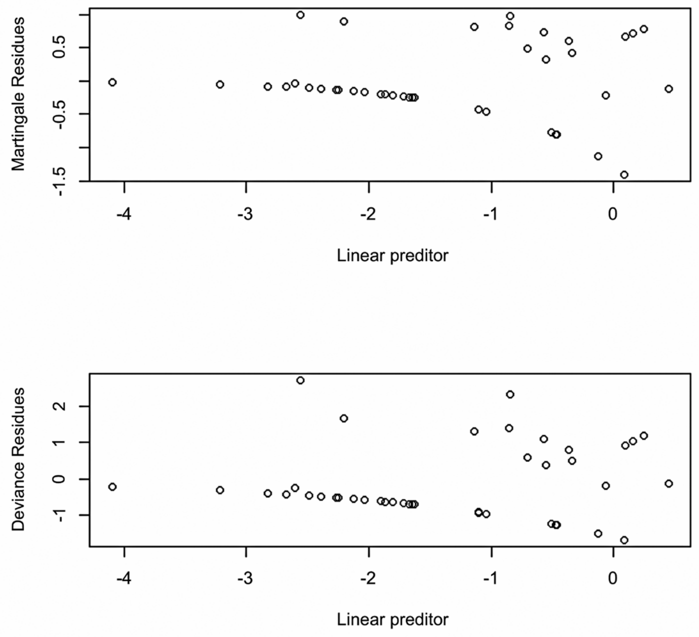

Figure 6S. Martingale and Deviance residues from the Cox model adjusted considering the level of the cytokine IL-17 in the dialysate
